# Iterative Rupture of the Patellar Tendon: A Case Report of an Original Technique for Revision Reconstruction Using an Adjustable Loop and an Artificial Ligament

**DOI:** 10.1155/2018/6107287

**Published:** 2018-09-17

**Authors:** N. Bouguennec, P. Colombet

**Affiliations:** Clinique du Sport de Bordeaux-Mérignac, 33700 Mérignac, France

## Abstract

Chronic rupture of the patellar tendon is a severe injury that leads to dramatic functional consequences including lack of extension and walking difficulty. Surgery is the gold standard to treat this type of injury, but revision reconstructions are problematic because an ipsilateral graft was often harvested for the initial surgery. Because fibrotic tissues on the patellar tendon need to be debrided, another graft must be added to reinforce the tendon. We reported the case of a former semiprofessional handball player, a 29-year-old man who presented an iterative rupture with the fracture of the transverse patellar tunnel 6 months after reconstruction using a semitendinosus graft and suture repair. We performed revision reconstruction surgery using an artificial ligament placed between the extensor mechanism and the tibia for extra-articular reinforcement to maintain extensor mechanism continuity. Two adjustable loops were also used to repair the patellar tendon tear. At 2-year follow-up, the patient was able to resume the practice of handball at a competitive level with good clinical and functional results. This technique can therefore be used as a salvage procedure for chronic iterative rupture of the patellar tendon.

## 1. Introduction

Rupture of the patellar tendon is a serious injury as it leads to lack of extension which interferes with activities of daily living [1]. For acute cases, suture repair is recommended, associated if needed with a graft for strengthening [2]. Chronic cases are more complicated to treat, and several different techniques have been described [2]. Reconstruction must be used to treat chronic ruptures as suture repair alone is insufficient. The gold standard includes debridement of the damaged tendon tissue coupled with the use of a graft. However, iterative rupture of chronic reconstruction can occur and is then a more challenging situation. We presented the case of a patient who underwent an initial reconstruction using an ipsilateral semitendinosus tendon (ST) with suture repair but who presented a reconstruction failure 13 months after primary surgery. Revision reconstruction using an artificial ligament and two adjustable loops was performed with good clinical and functional outcomes at 2-year follow-up.

## 2. Case Report

**Figure 1:**
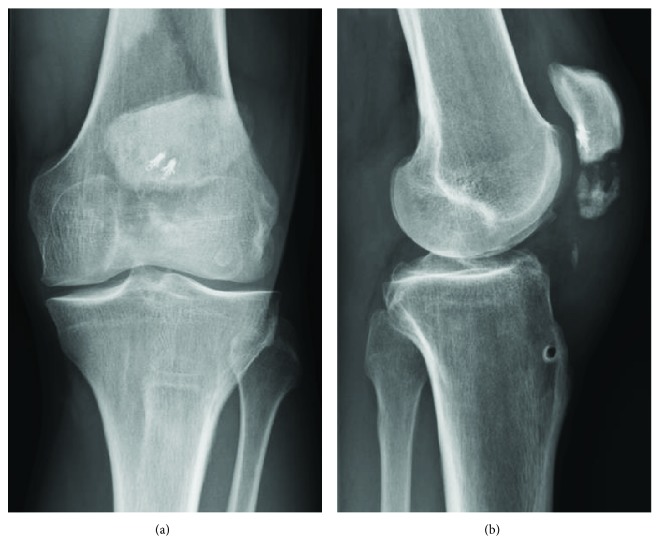
(a) Anteroposterior and (b) sagittal preoperative X-rays before the revision surgery. The iterative rupture of the extensor mechanism is explained by the fracture of the transverse patellar tunnel drilled during the initial surgery to pass the semitendinosus graft.

A 29-year-old man, a former semiprofessional handball player, had a traumatic rupture of the proximal side of the patellar tendon of the left knee in 2016 during a match. No associated disease was reported. Primary surgery was performed in another health facility through a median approach using 2 anchors for tendon repair protected by an additional ipsilateral semitendinosus graft (patellar and tibial tunnels). The patient came to our health facility following severe functional deficits after an iterative rupture without having experienced any new trauma 13 months after the initial surgery. The iterative rupture of the knee extensor mechanism was also an iatrogenic fracture of the transverse patellar tunnel (Figure 1). Clinically, walking was not possible, there was a lack of active extension and hemarthrosis with pain. There were no scar problems, no signs of deep or superficial infection, and no cutaneous wound. A huge gap was clinically observed between the patella and the patellar tendon. Considering the patient's age, his preinjury sports level, and lack of active extension, the decision was made to perform a revision procedure. An artificial ligament (LARS®) and two adjustable loops, free ends of the PULLUP® BTB (SBM SAS, France), were used to enhance the patellar tendon repair.

### 2.1. Technique

**Figure 2:**
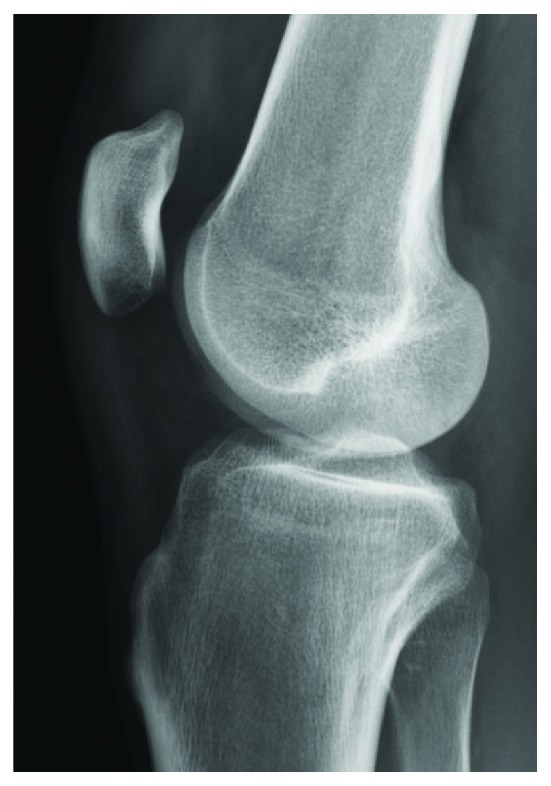
Preoperative X-ray of the contralateral knee to measure the Caton-Deschamps index and patellar height.

A preoperative lateral standard X-ray was taken of the contralateral knee at 30° of flexion to measure the Caton-Deschamps index and patellar height (Figure 2). The patient was placed in a supine position under general anesthesia with a tourniquet at the proximal part of the thigh. The previous median approach was used. The patellar fracture and the site of the previous rupture were cleaned to remove fibrous tissue and hematoma. Previous anchors were left in place.

**Figure 3:**
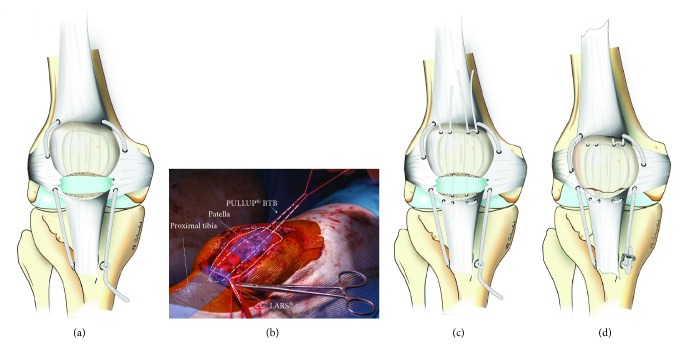
Pictures of the surgical steps. (a) With the artificial ligament in place. (b) Perioperative view with the artificial ligament and one adjustable loop. (c) With the artificial ligament and the two adjustable loops. (d) The final step when the artificial ligament and the loops are tightened. A staple is placed to secure the artificial ligament.

**Figure 4:**
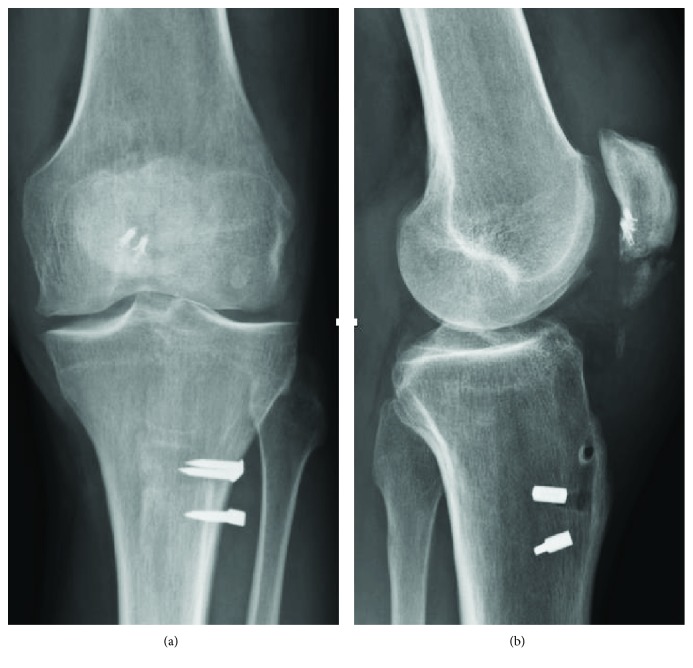
(a) Anteroposterior and (b) sagittal postoperative X-rays at 3 months.

The first step was to place the ligament advanced reinforcement system (LARS® polyethylene terephthalate fibers 6 mm ref. L030307 ACFAR 32 CK). A new transverse tunnel was drilled in the tibia, distally to the tibial tunnel of the initial surgery, with a 5.5 mm drill, and the LARS® was inserted in the tibial tunnel. The artificial ligament was then passed through the lateral retinaculum and above the patella at the junction with the quadriceps tendon in a Pulvertaft manner and through the medial retinaculum to return to its origin (Figure 3(a)). Two longitudinal tunnels were drilled in the patella using a 2.4 mm drill. The loops of a PULLUP® BTB (the plate was removed from the device) were first passed into the patellar tendon and then into the patella through the two longitudinal tunnels using a shuttle relay. Next, the free ends were pulled down in the opposite patellar tunnels. At the proximal side of the reconstruction, the two free ends of the PULLUP® BTB were inserted into each braid to close the system (Figures 3(b) and 3(c)). The distal and medial ends of the LARS® were tightened with a clamp in order to restore normal patellar height and secured with 2 staples. Then, the 2 PULLUP® BTB loops were tightened (Figure 3(d)). The previous tendon rupture was closed and reinforced with separate X-knots using absorbable sutures. The skin was closed. The knee was placed in an articulated brace with compressive ice therapy for 24 hours, and a postoperative X-ray was taken (Figure 4).

### 2.2. Follow-Up

**Figure 5:**
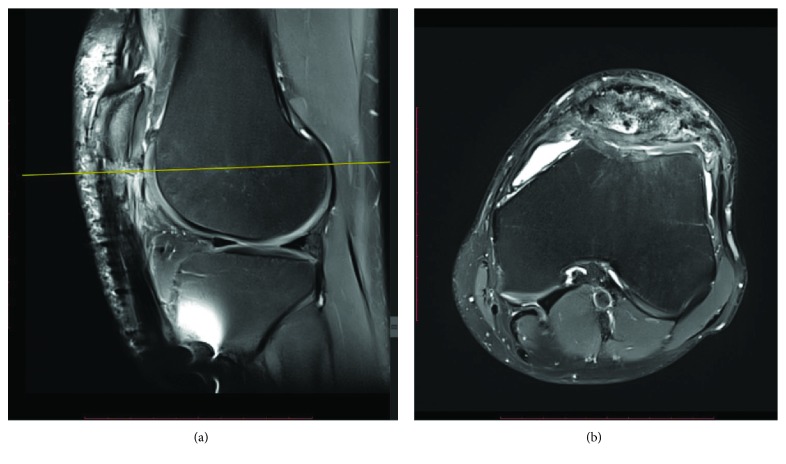
(a) T2 sagittal slice and (b) T2 axial slices of the control MRI at 1-year follow-up. The axial slice corresponds to the level of the fracture of the transverse patellar tunnel. Fracture is healed and continuity of the patellar tendon is preserved.

For postoperative care, weight bearing was not allowed for 6 weeks and mobilization of the knee was immediately started between 0 and 45° for 3 weeks then from 0 to 90° from 3 to 6 weeks. No complication was reported during the postoperative period. At 3 months, the patient was pain free and could walk without the aid of crutches. He was able to resume handball practice at 6 months after a control MRI. At one-year follow-up, he was able to play handball with complete knee extension strength (compared with the contralateral side) and was able to return to a semiprofessional level. The range of motion of the knee was 0-0-130°. The MRI at 1 year showed complete healing of the patellar tendon and the bone (Figure 5).

## 3. Discussion

For Garner et al., patellar tendon rupture represents 12% of the extensor mechanism injuries [1]. They emphasize that this type of injury is not so uncommon and has dramatic consequences on daily life as it interferes with the patient's ability to stand up and walk. It becomes an even rarer injury when a chronic iterative rupture occurs secondly to a misdiagnosis or to a second rupture. We reported the case of a patient with an iterative rupture of the extensor mechanism, without rerupture of the tendon, but with a fracture of the transverse patellar tunnel drilled during the initial surgery. The initial rupture occurred at the inferior pole of the patella which is the most common site of rupture of the patellar tendon [2]. The second rupture was of the transverse patellar tunnel drilled during the initial surgery, which is a typical complication that has been extensively described [3]. The original technique described in this article can also be performed to treat chronic rupture of the proximal part of the patellar tendon.

Chronic rupture of the patellar tendon is a challenging situation as debridement of the tendon that is required prior to reconstruction can disrupt extensor mechanism continuity. Additional reinforcement is the recommended, gold standard technique [2]. Numerous techniques involving a cerclage wire or graft have been described. Though allografts can be used [4, 5], they expose patients to the risk of infectious disease transmission and are currently difficult to obtain in France. Autografts are the most common choice, especially the hamstring tendons, of which the semitendinosus is the most extensively described because it can be used to augment the suture repair of the patellar tendon and is easy to harvest through the same approach [2, 6–8]. The use of the hamstring tendons can also be useful in case of rupture of the patellar tendon with total knee replacement [9]. Another augmentation technique uses the contralateral patellar tendon, but this technique can weaken the contralateral healthy knee [10, 11]. In our case, the ipsilateral ST graft was used for the initial surgery to obtain a frame structure when passed through the tunnels in the tibia and the patella. Since rerupture involved the fracture of the patellar tunnel, a contralateral bone-patellar tendon-bone graft was not feasible as a socket could not be drilled in the fractured patella. We therefore decided to use an artificial ligament which was passed above the patella to avoid fragilizing it even more. Artificial ligaments have already been used to reconstruct chronic extensor mechanism rupture, and Talia and Tran described a technique with the same device (LARS®) to reinforce the patellar tendon reconstruction with a good functional outcome, a range of motion of 0–130°, and no sign of synovitis [12]. They did not use any graft to reinforce the reconstruction. Naim et al. used two bundles of LARS® to reconstruct the patellar tendon for a chronic rupture with a full flexion and full extension strength at one-year follow-up [13]. Gilmore et al. did not report any complications with the use of an artificial graft [2].

In our case, to reinforce the reconstruction, two adjustable loops were added (two PULLUP® BTB, SBM SAS, France), from which the buttons were removed, and were passed through the patellar tendon and through the patella to progressively adapt the length of the tendon to the Caton-Deschamps index which was assessed preoperatively. Two longitudinal tunnels were drilled in the patella at a diameter of only 2.4 mm to limit the risk for an additional fracture. No articles describing the use of this device could be found in the literature.

Postoperative rehabilitation and immobilization (cast, hinged brace, or nothing) are also debated, and very few studies were found in the literature [2] with a small number of patients. There is currently no compelling evidence to advocate a certain type of immobilization or postoperative weight-bearing recommendation. As walking requires contraction of the quadriceps, it appeared logical to us not to allow initial weight bearing and to progressively increase the range of motion. Regarding range of motion recovery, we did not find any recommendations in the literature either.

There are a few clinical studies with follow-up after chronic patellar tendon ruptures [2, 14], but the outcomes in chronic cases are worse than in acute cases. Maffuli et al. [14] reported a postoperative mean flexion of 132°, and 62% of patients were able to return to the same level of sport. In this case, we also found a good functional outcome with complete flexion (130°) of the knee at the final follow-up and the patient was able to return to his professional level of sport at one year.

## 4. Conclusion

We presented an original technique to reconstruct the extensor mechanism after rerupture when the ST has ever been harvested and when the contralateral bone-patellar tendon-bone graft cannot be used. In this case, it was also an iatrogenic fracture of the distal part of the patella. Good clinical and functional results were obtained as the patient was able to go back to the same level of sport.
